# Comparative Imaging of Allergic Fungal Sinusitis With Orbital and Intracranial Extension: A Case Report

**DOI:** 10.7759/cureus.111057

**Published:** 2026-06-17

**Authors:** Jennifer Adams, Gregory Wrubel, Mehmet Albayram

**Affiliations:** 1 Radiology, AdventHealth Florida Hospital, Orlando, USA; 2 Neuroadiology, AdventHealth Florida Hospital, Orlando, USA; 3 Neuroradiology, AdventHealth Florida Hospital, Orlando, USA

**Keywords:** allergic fungal sinusitis, intracranial extension, orbital apex compression, paranasal sinus disease, radiologic grading system

## Abstract

Allergic fungal sinusitis (AFS) is classified as a non-invasive, immunologically mediated chronic rhinosinusitis. It can rarely mimic aggressive infectious or neoplastic disease. We present two companion cases demonstrating orbital apex compression without intracranial involvement and another case with epidural extension, respectively. These cases challenge the conventional understanding of AFS and emphasize the importance of advanced imaging. We propose a novel AFS aggression spectrum (AAS) to stratify disease severity and categorize the progressive imaging features of allergic fungal sinus disease within a four-tier diagnostic framework.

## Introduction

Allergic fungal sinusitis (AFS) is a subtype of chronic rhinosinusitis characterized by eosinophilic mucin and fungal colonization without tissue invasion, most commonly seen in immunocompetent individuals [1-2]. The Bent and Kuhn criteria remain the most widely accepted diagnostic framework for AFS [2-4]. These criteria include evidence of type I hypersensitivity, nasal polyposis, characteristic radiographic findings such as hyperdense sinus contents, eosinophilic mucin without fungal invasion, and positive fungal staining [3-4]. While this classification has been instrumental in identifying AFS, it labels the disease as either present or absent, without accounting for disease progression, complications, or surgical urgency.

These limitations become increasingly important in cases where AFS presents with orbital or intracranial complications. Orbital and intracranial extension, reported in up to 10%-15% of advanced cases, represent significant complications of AFS and are associated with increased risk of visual loss and neurologic morbidity [5-6]. To address this gap, we propose an AFS aggression spectrum (AAS) to reflect the continuum of AFS severity. This proposed AFS grading system draws elements from both the Lund-Mackay CT score [5], by quantifying sinus burden and anatomic involvement, and the Chandler classification of orbital complications [6]. Such existing diagnostic frameworks' criteria focus on disease identification rather than severity stratification. These frameworks inform our proposed severity model, which integrates disease extent with complication risk to offer improved predictive and management utility for AFS.

## Case presentation

We present two distinct patients demonstrating the spectrum of AFS, ranging from pediatric orbital apex compression to intracranial epidural extension. A comparison of established diagnostic frameworks and the proposed AAS is summarized in Table 1.

**Table 1:** Comparison of established diagnostic and classification systems in allergic fungal sinusitis (AFS). AAS, AFS aggression spectrum; CT, computed tomography

Classification system	Purpose	Components	Limitation
Bent and Kuhn [3]	Diagnostic	Hypersensitivity, nasal polyps, hyperdense CT, eosinophilic mucin, fungal stain	Binary, no severity grading
Lund and Kennedy [5]	Disease burden	CT opacification scoring	No complication stratification
Chandler et al. [6]	Orbital complications	Stages I-V orbital involvement	Limited to orbital disease
AAS	Severity + risk	Imaging-based progression (Grades I-IV)	Requires large-cohort validation

**Figure 1:**
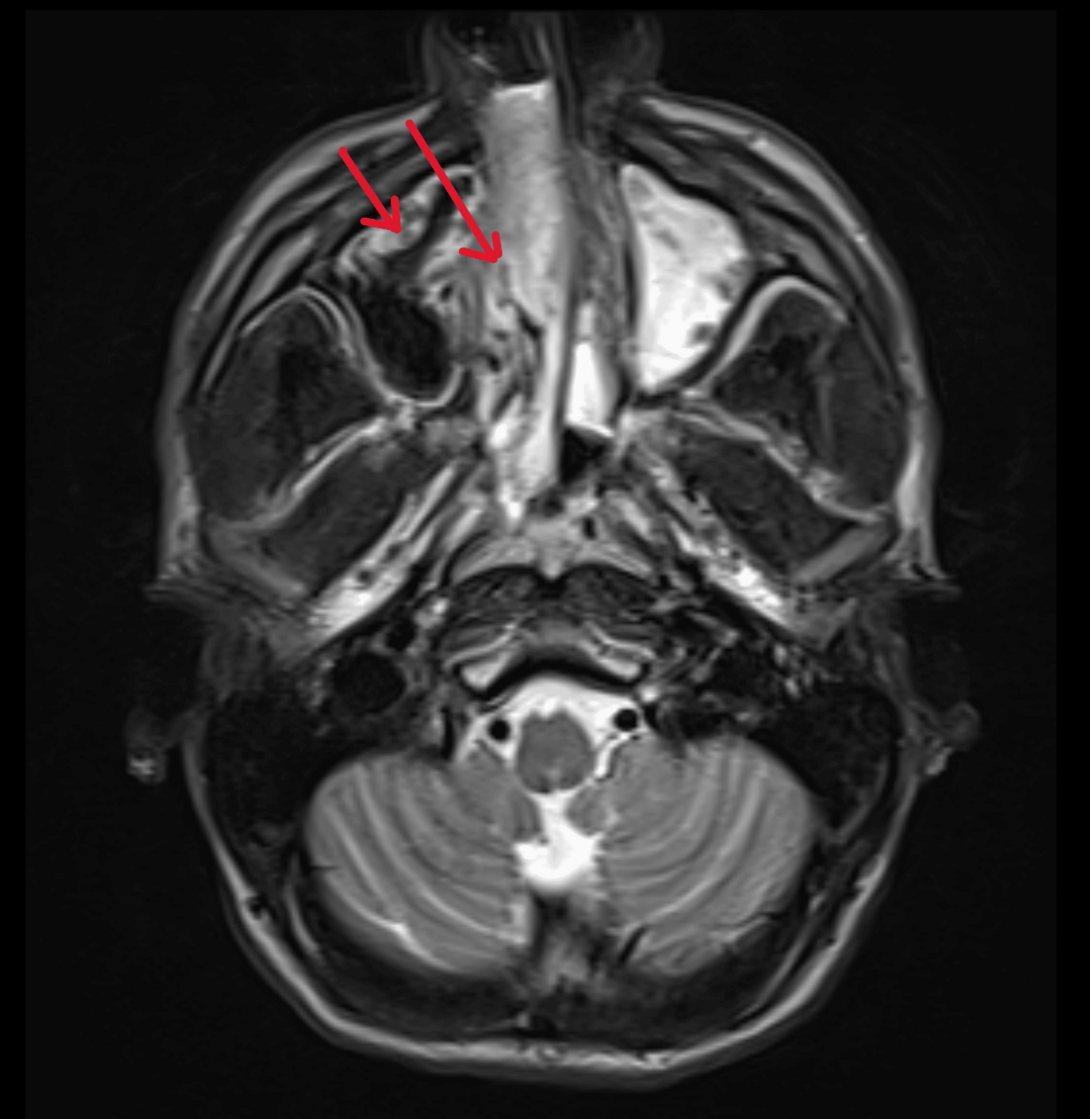
Axial T2-weighted MR image demonstrating marked T2 signal dropout (red arrows) within the sinonasal contents, consistent with dense fungal secretions. MR, magnetic resonance

**Figure 2:**
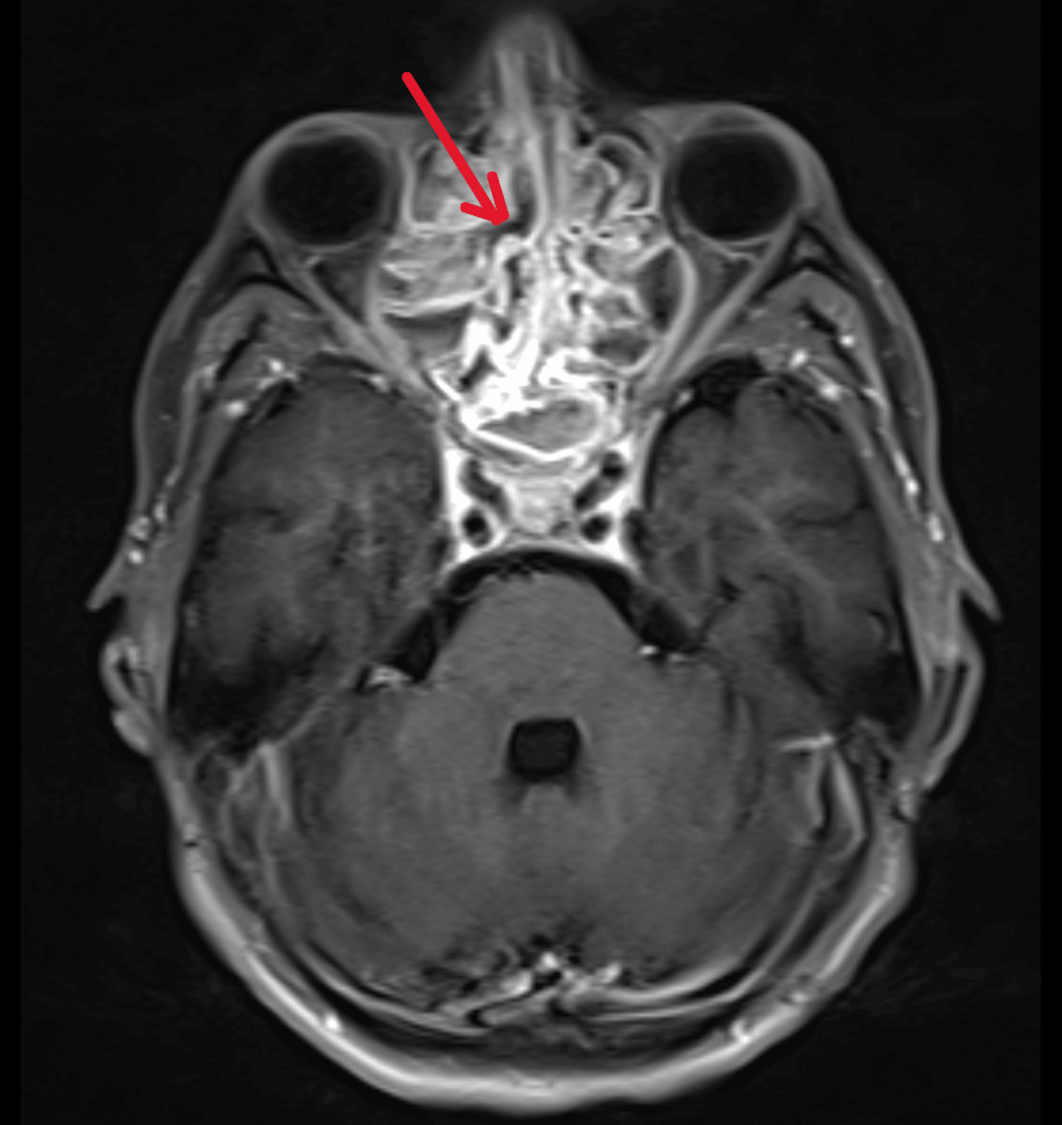
MR T1 sequence, axial view, demonstrating isointense T1 signal within the sinonasal contents and sinonasal mucosal enhancement following contrast administration (red arrow). MR, magnetic resonance

Patient 1 was a 17-year-old immunocompetent male with asthma, eczema, and allergic rhinitis who presented with bilateral eye pain and mild proptosis. Neurologic examination, extraocular movements, and visual acuity were normal. MRI of the orbits and paranasal sinuses demonstrated marked T2 signal dropout of the sinonasal contents corresponding to dense fungal concretions (Figure 1), with isointense-to-hyperintense signal on T1-weighted imaging and thin peripheral mucosal enhancement after contrast administration (Figure 2).

No orbital or intracranial extension was identified. CT confirmed hyperdense inspissated sinus contents with sinus expansion, smooth wall thinning, and focal dehiscence. Notably, bilateral lamina papyracea erosion with medial rectus displacement resulted in mass effect on both optic nerves, without findings of invasive fungal disease such as aggressive bone loss, peri-antral fat infiltration, or cavernous sinus involvement. The diagnosis of AFS with bilateral orbital apex compression was favored. The patient was managed with a short corticosteroid taper and intranasal steroids, with coordinated ENT and ophthalmology follow-up. Given preserved vision and absence of invasive features, planned outpatient endoscopic sinus decompression was pursued. The imaging findings and the patient’s atopic history and characteristic radiologic features fulfill the Bent and Kuhn diagnostic criteria for AFS.

**Figure 3:**
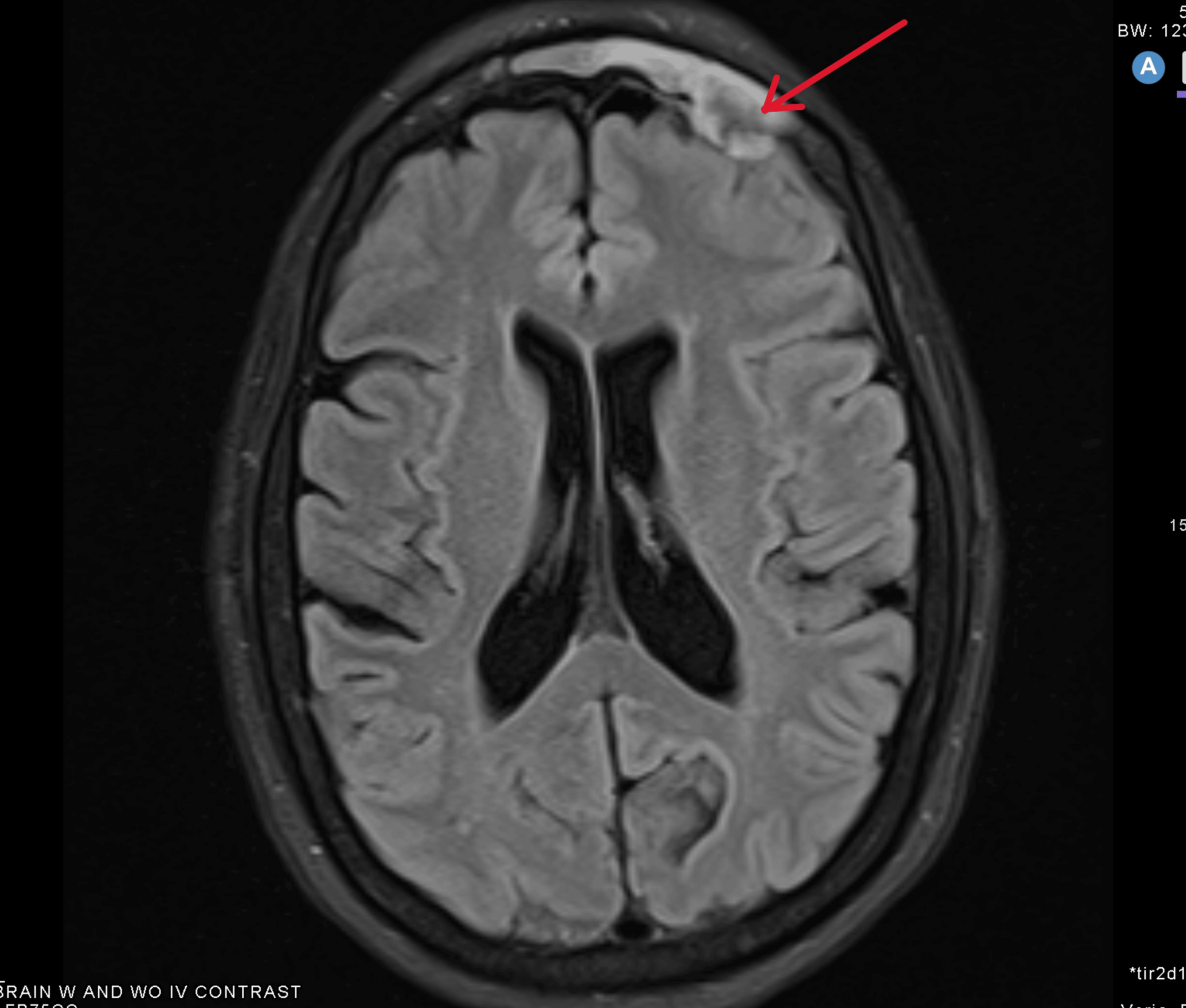
Axial T2-weighted fluid-attenuated inversion recovery (FLAIR) magnetic resonance (MR) image demonstrating a 1.1-cm epidural collection with increased signal intensity (red arrow) along the anterior cranial fossa.

**Figure 4:**
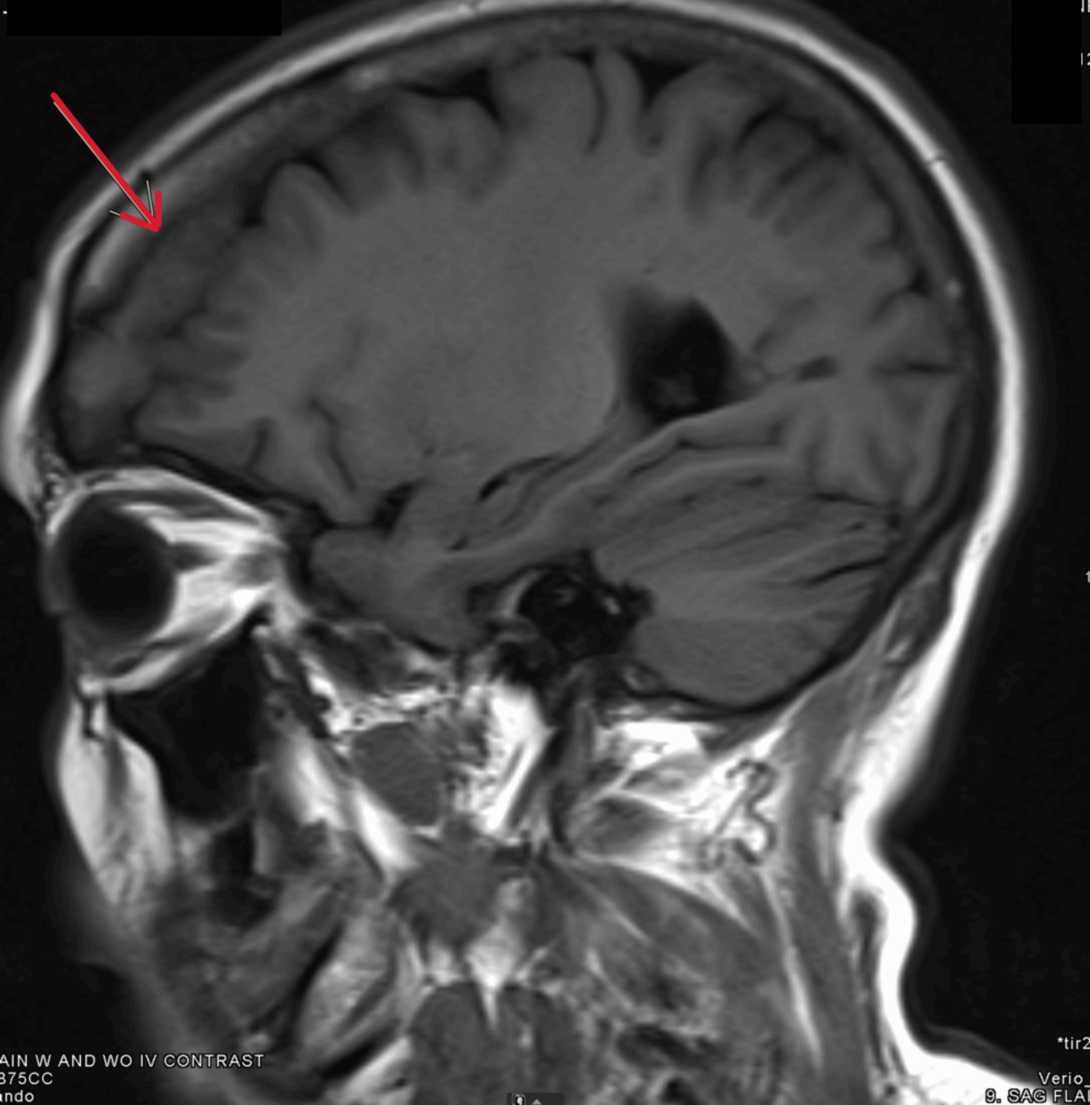
MR T1 FLAIR sequence, sagittal view, demonstrating isointense extradural intracranial extension of allergic fungal material along the anterior cranial fossa (red arrow). FLAIR, fluid-attenuated inversion recovery; MR, magnetic resonance

Patient 2 was a 61-year-old woman with a history of chronic sinusitis, hypertension, and seizure disorder who presented with progressive headache, vomiting, and unilateral photophobia. MRI revealed a 1.1-cm epidural collection along the left anterior cranial fossa that was isointense on T1-weighted imaging, with thin peripheral dural enhancement. The lesion demonstrated no restricted diffusion, effectively excluding a pyogenic abscess (Figures 3-4).

Diffuse T2 hypointensity within the opacified frontal and ethmoid sinuses suggested dense fungal concretions. CT demonstrated complete left frontal sinus opacification, marked hyperdensity of intraluminal material, and focal dehiscence of the posterior frontal sinus wall. The frontal sinus contents were seen extending into the epidural space and abutting the left frontal lobe without cerebritis or parenchymal invasion. The imaging appearance raised concern for advanced AFS with epidural extension. Other differential considerations, including fungal abscess, mucocele with secondary infection, sinonasal lymphoma, and fungal mycetoma, were considered less likely based on the absence of restricted diffusion, lack of a discrete cystic mass, absence of a homogeneously enhancing soft-tissue component, and the presence of widespread hyperdense allergic mucin.

Given intracranial extension and mass effect, the patient underwent urgent combined neurosurgical and otolaryngologic intervention, including left frontal craniotomy for epidural evacuation and comprehensive functional endoscopic sinus surgery. She was transitioned to oral antifungal therapy with a favorable clinical response. Postoperative antifungal therapy was administered as adjunctive treatment given the extensive disease burden and intracranial extension, although AFS is classically noninvasive. Similarly, the imaging pattern, clinical presentation, and absence of true invasive features are consistent with noninvasive AFS as defined by Bent and Kuhn criteria.

## Discussion

The two companion cases illustrate the broad spectrum of AFS and its ability to mimic invasive sinonasal pathology despite its noninvasive histopathology. Both patients demonstrated the hallmark imaging features of AFS, including sinonasal hyperdensity on CT, marked T2 hypointensity due to desiccated fungal concretions, and peripheral mucosal enhancement on post-contrast MRI. These imaging findings are well established in the literature and help differentiate AFS from invasive fungal sinusitis. These findings highlight that the morbidity of AFS is driven not by tissue invasion but by progressive expansile remodeling and mass effect on adjacent critical structures.

Patient 1 demonstrates how expansile allergic mucin may produce clinically significant orbital apex compression without direct soft tissue invasion. Several published cases parallel the orbital apex involvement seen in Patient 1. Allami et al. described young immunocompetent patients presenting with acute visual loss and CT evidence of extensive bony remodeling, lamina papyracea erosion, and sphenoid involvement resulting in optic nerve compression [7]. Unfortunately, vision did not recover despite surgical decompression, emphasizing the time-sensitive nature of this process. Tong et al. reported advanced AFS with bilateral vision loss and orbital extension, emphasizing that optic neuropathy may result from mechanical compression, vascular compromise, or perineural inflammation in the absence of invasion [8]. Gupta et al. further proposed a dual mechanism of injury, combining pressure-related optic nerve compromise with a local immunologic response to fungal antigens, which may explain disproportionate visual dysfunction relative to the degree of bony encroachment [9]. A larger series by Rowan et al. confirms that lamina papyracea dehiscence and orbital extension represent the typical imaging spectrum of AFS, with adult patients more frequently demonstrating bilateral expansile disease [10]. Collectively, these studies support the conclusion that smooth expansile remodeling and focal dehiscence alone are sufficient to threaten optic nerve integrity even in noninvasive disease.

Patient 2 demonstrates a more advanced manifestation of AFS, with extradural intracranial extension through posterior frontal sinus wall dehiscence. The epidural collection was T1 isointense with thin peripheral enhancement and lacked restricted diffusion, findings that favor inflammatory allergic mucin rather than a pyogenic abscess. The extradural intracranial extension demonstrated in Patient 2 was a less common manifestation of AFS, reflecting chronic expansile remodeling rather than invasive disease. Kinsella et al. identified a subset of AFS patients with skull base erosion and extradural intracranial involvement, all meeting histopathologic criteria for noninvasive disease and successfully managed with endoscopic approaches alone [11]. Similarly, Ikram et al. reported a series of 26 patients with intracranial extradural extension, all immunocompetent and without tissue invasion, reinforcing that this presentation represents mass effect rather than true fungal invasion [12]. Aribandi and Bazan further demonstrated that intracranial AFS retains its characteristic CT and MRI signature, including central hyperdensity and T2 hypointensity, even when extending beyond the sinuses [13]. Importantly, these cases consistently respond to surgical debridement without the need for neurosurgical intervention, highlighting that recognition of this pattern is critical to avoid misclassification as invasive disease. Postoperative antifungal therapy was administered as adjunctive treatment in the setting of extensive disease and intracranial extension, although AFS is classically noninvasive and management remains primarily surgical with corticosteroid therapy.

Across both cases and the supporting literature, AFS has a characteristic imaging signature regardless of disease extent. Hyperdense sinus contents on CT, T2 signal void on MRI, smooth bony expansion, and peripheral mucosal enhancement are the most reliable diagnostic features. Radiographic bone erosion alone does not imply invasive fungal disease, a distinction emphasized by Bent and Kuhn in their original diagnostic framework [3]. The absence of diffusion restriction, lack of enhancing soft tissue invasion, and preservation of surrounding fat planes help differentiate advanced noninvasive AFS from invasive fungal sinusitis.

We propose the AAS, a novel imaging-based framework that stratifies disease severity according to progressive radiologic findings. Grade I disease includes hyperdense sinonasal contents without significant expansion or complications. Grade II disease demonstrates smooth bony remodeling and early orbital encroachment. Grade III disease includes bone erosion with optic nerve compression or orbital apex crowding, representing vision-threatening disease that requires urgent surgical decompression. Grade IV disease demonstrates intracranial extension, typically extradural, with associated dural involvement necessitating multidisciplinary management. Unlike existing diagnostic criteria, the AAS incorporates progressive imaging severity and anticipates potential complications. A summary of AAS, including imaging findings, associated complications, and management implications, is provided in Table 2

**Table 2:** AFS aggression spectrum (AAS): Imaging-based grading, complications, and management framework. AFS, allergic fungal sinusitis

Stage	Imaging findings	Complications	Clinical risk	Management
I	Hyperdense sinus contents, T2 hypointensity, no expansion	None	Low	Medical therapy
II	Sinus expansion, bony thinning, and early orbital encroachment	Mild orbital mass effect	Moderate	ENT referral, planned FESS
III	Bone erosion, orbital apex crowding, and optic nerve compression	Vision-threatening	High	Urgent decompression
IV	Intracranial extradural extension and dural enhancement	Intracranial mass effect	Critical	ENT + neurosurgery

Application of the AAS framework to these companion cases demonstrates its clinical utility. Patient 1 is best classified as Grade III disease due to orbital apex crowding, lamina papyracea erosion, and optic nerve impingement without intracranial spread. Patient 2 meets criteria for Grade IV disease because of extradural intracranial extension with epidural inflammatory phlegmon and dural enhancement. These cases demonstrate how imaging findings may be risk-stratified and can guide the urgency of intervention. To our knowledge, no prior imaging-based grading system has formally integrated radiologic markers of AFS into a tiered framework. The AAS framework should be considered preliminary and requires prospective validation in larger cohorts.

## Conclusions

These companion cases demonstrate that even in immunocompetent patients, AFS can mimic invasive disease and cause serious complications. Early recognition of red flags, such as hyperdense sinus contents, bone erosion, and dural enhancement with non-enhancing, non-diffusion-restricting epidural material, is essential. The AAS offers a novel framework for stratifying disease severity and guiding multidisciplinary management. By integrating imaging findings into clinical decision-making, the AAS enhances diagnostic accuracy and facilitates timely intervention.
